# Development and validation of a 90-day mortality prediction model following endobiliary drainage in patients with unresectable malignant biliary obstruction

**DOI:** 10.3389/fonc.2022.922386

**Published:** 2022-09-06

**Authors:** Panotpol Termsinsuk, Phunchai Charatcharoenwitthaya, Nonthalee Pausawasdi

**Affiliations:** ^1^ Siriraj GI Endoscopy Center, Faculty of Medicine Siriraj Hospital, Mahidol University, Bangkok, Thailand; ^2^ Gastroenterology Unit, School of Medicine, Institute of Medicine, Suranaree University of Technology, Nakhon Ratchasima, Thailand; ^3^ Division of Gastroenterology, Department of Medicine, Faculty of Medicine Siriraj Hospital, Mahidol University, Bangkok, Thailand

**Keywords:** obstructive jaundice, malignant bile duct obstruction, endoscopic retrograde cholangiopancreatography - ERCP, biliary drainage, stent, mortality, prediction model

## Abstract

**Background:**

Palliative endobiliary drainage is the mainstay treatment for unresectable malignant biliary obstruction (MBO). Despite optimal drainage, the survival benefit is arguable. This study aimed to identify factors predicting post-endoscopic drainage mortality and develop and validate a mortality prediction model.

**Methods:**

We retrospectively analyzed data for 451 patients with unresectable pancreatobiliary cancers undergoing first endoscopic retrograde cholangiopancreatography (ERCP)-guided endobiliary stent placement between 2007 and 2017. We randomly assigned patients in a 3:1 fashion into a derivation cohort (n=339) and validation cohort (n=112). Predictors for 90-day mortality post-stenting were identified from the derivation cohort. A prediction model was subsequently developed and verified with the validation cohort.

**Results:**

The overall 90-day mortality rate of the derivation cohort was 46.9%, and the mean age was 64.2 years. The 2 most common diagnoses were cholangiocarcinoma (53.4%) and pancreatic cancer (35.4%). In all, 34.2% had liver metastasis. The median total bilirubin (TB) level was 19.2 mg/dL, and the mean serum albumin was 3.2 g/dL. A metallic stent was used for 64.6% of the patients, and the median stent patency time was 63 days. A total of 70.8% had TB improvement of more than 50% within 2 weeks after stenting, and 14.5% were eligible for chemotherapy. Intrahepatic obstruction (OR=5.69; *P*=0.023), stage IV cancer (OR=3.01; *P*=0.001), pre-endoscopic serum albumin (OR=0.48; *P*=0.001), TB improvement within 2 weeks after stenting (OR=0.57; *P*=0.036), and chemotherapy after ERCP (OR=0.11; *P*<0.001) were associated with 90-day mortality after stenting. The prediction model was developed to identify the risk of death within 90 days post-stent placement. The AUROC was 0.76 and 0.75 in derivation and validation cohorts. Patients with a score ≥ 1.40 had a high likelihood of death, whereas those scoring < -1.50 had a low likelihood of death. Additionally, a score ≥ 0.58 provided a 75.2% probability of death, which highlights the usability of the model.

**Conclusions:**

This study proposes a useful validated prediction model to forecast the 90-day mortality of unresectable MBO patients after stenting. The model permits physicians to stratify the death risk and may be helpful to provide a proper palliative strategy.

## Introduction

Malignant biliary obstruction (MBO) is a common cause of obstructive jaundice worldwide. Cholangiocarcinoma, pancreatic cancer, gallbladder cancer, and metastatic lymph nodes comprise the majority of MBOs (1). Patients with MBO usually present with an advanced and unresectable stage at diagnosis (2). The symptoms (jaundice, pruritus, malaise, anorexia, and weight loss) are unpleasant and impair patients’ quality of life (2–5). Furthermore, due to pre-existing jaundice, initiation of chemotherapy is usually delayed to avoid the risk of hepatotoxicity (6–8). Consequently, the prognosis of unresectable MBO patients is generally poor, and the 5-year survival rate is usually less than 5% (1, 9).

Endoscopic retrograde cholangiopancreatography (ERCP)-guided endobiliary drainage with stent placement is a primary treatment for unresectable MBO patients to relieve symptoms and provide the opportunity for chemotherapy (10–12). In addition to the higher technical success rate of 90%–95%, ERCP-guided endobiliary stent placement demonstrates reduced adverse events, hospitalization, and total costs. Additionally, it is associated with more prolonged survival than percutaneous transhepatic biliary drainage and surgical drainage (13–18). However, 10% of unresectable MBO patients do not respond to endobiliary stents, and survival after stent placement eventually worsens (19).

Various clinical studies have explored the predictors for mortality after endoscopic endobiliary stent placement. Prat et al. demonstrated that a tumor size greater than 3 cm was associated with shorter post-stenting survival than a tumor size less than 3 cm (20). The presence of liver metastasis was the major contributor to 24-week mortality and was significantly associated with shorter survival after endobiliary drainage in patients with unresectable MBO (21–23). For pre-endoscopic laboratories, serum levels of total bilirubin (TB) greater than 14 mg/dL and albumin levels lower than 2 mg/dL were associated with 30-day mortality after endobiliary stenting in MBO patients (24, 25). However, pre-endoscopic white blood cell counts lower than 11 000/mm^3^ and serum gamma-glutamyl transferase (GGT) lower than 165 U/L were associated with better survival outcomes in unresectable pancreatic cancer patients (26).

Nevertheless, clinical studies identifying potential predictors for post-stenting and mortality are limited and generally have small sample sizes. Moreover, other clinical predictors that could influence the mortality outcome, including the site of obstruction and radiographic findings, have not been established by prior studies.

The present study explored the clinical predictors for 90-day mortality after endoscopic endobiliary stent placement. A prediction model for 90-day mortality was also developed and internally validated to identify suitable unresectable MBO patients to proceed with optimal palliation according to their predicted post-stenting mortality.

## Materials and methods

### Study population

The Institutional Review Board of the Faculty of Medicine Siriraj Hospital approved the study protocol, which met the ethical guidelines of the 1975 Helsinki Declaration (approval number 097/2019). A retrospective review of the ERCP database of a large tertiary care center was conducted. Eligible patients were those who underwent their first ERCP with endobiliary stent placement for unresectable MBO due to pancreatobiliary cancers between January 2007 and December 2017. The criteria for unresectable MBO were as follows:

- Inoperable locally advanced disease due to major vascular involvement, bilateral or contralateral portal vein, hepatic artery, or secondary biliary radicle involvement
- Bismuth–Corlette type IV hilar cholangiocarcinoma
- Distant organ or lymph node metastasis
- Unfit for surgery due to significant comorbid conditions and poor functional status.

A total of 1334 patients with unresectable MBO were identified. Of these, 883 patients were excluded. The reasons were incomplete data (n=417), biliary obstruction caused by malignancies other than pancreatobiliary cancers (n=60), concomitant other biliary drainage procedures (n=128), previous endobiliary stent placement (n=73), failed cannulation or no stent placement (n=64), advanced cirrhosis (n=62), death within 2 weeks after endobiliary stent placement (n=30), absence of jaundice (n=25), receiving chemotherapy prior to stenting (n=18), and percutaneous cholecystostomy (n=6). The remaining 451 patients were enrolled and randomly divided in a 3:1 fashion into a “derivation cohort” (n=339) and a “validation cohort” (n=112) using computer generation by simple randomization method.

All relevant clinical and laboratory data before and after ERCP were collected. In addition, cross-sectional images and cholangiographic findings were reviewed. The diagnosis of malignant obstruction was based on either histopathology alone or combined imaging and clinical follow-up in cases where histology could not be obtained.

### Statistical analysis

Categorical variables are expressed as numbers and percentages. Continuous variables are expressed as the mean ± standard deviation for normally distributed data and the median with interquartile range (IQR) for skewed distribution data. Groups were compared using the Chi^2^ test or Fisher’s exact test for categorical variables and the independent *t*-test or Mann–Whitney U test for continuous variables. Differences were considered statistically significant if the probability (*P)* values were < 0.05.

Using univariate logistic regression, clinical variables that influenced the study outcomes were identified and compared between surviving and non-surviving patients. The odds ratios (ORs) and 95% confidence intervals (CIs) were obtained. The significant variables from this step were entered into a multivariate logistic regression model as demonstrated in Model 1. Because of the long recruitment period of the study, the calendar year of endoscopic procedures was considered to control the potential effect on the outcomes in the logistic regression models as shown in Model 2. The coefficients estimated for each factor, which provided the best predictive ability, were relatively weighted to compute the mortality prediction model. The calibration of the mortality prediction model was assessed by comparing the actual observed risk and the average probability of death predicted by the score. The Hosmer–Lemeshow test was used to assess the corresponding goodness-of-fit.

The discriminative power of the developed prediction model was estimated by calculating the area under a receiver operating characteristic curve (AUROC). The sensitivity, specificity, positive predictive value (PPV), negative predictive value (NPV), positive likelihood ratio (LR+), and negative likelihood ratio (LR−) were also determined. Based on a receiver operating curve, cutoffs were selected to categorize patients into a low and high probability (~90%) of death within 90 days after endobiliary stenting. A Cox proportional hazards model was used to estimate the hazard ratio and probability of death according to quartiles of probability score. The overall cumulative survival was estimated using the Kaplan–Meier method. All statistical analyses were performed using PASW Statistics for Windows, version 18 (SPSS Inc., Chicago, IL, USA).

## Results

### Baseline characteristics

Of 451 patients with unresectable pancreatobiliary cancers who underwent their first ERCP-guided endobiliary stent placement, 339 and 112 were randomly assigned to the derivation and validation cohorts, respectively. The mean age of the derivation cohort was 64.2 ± 12.4 years, and 173 patients (51.0%) were men. Cholangiocarcinoma was the most common diagnosis, accounting for 53.4% of cases, followed by pancreatic cancer (35.4%), gallbladder cancer (10.0%), and malignant intrapapillary mucinous neoplasm (1.2%). Liver metastasis was noted in 34.2% of patients. The most common clinical presentations were jaundice (89.4%), weight loss (63.7%), abdominal pain (57.2%), and fever (7.1%). Acute cholangitis was diagnosed in 18.0% of cases at the initial presentation. The median pre-endoscopic TB level was 19.2 ± 9.5 mg/dL, and the mean serum albumin was 3.2 ± 0.6 g/dL. Thirty-nine percent of patients had hilar obstruction, whereas 56.6% had an extrahepatic obstruction detected on imaging. Evidence of advanced-stage disease was present: major vascular invasion (47.5%), portal vein invasion (28.3%), distant metastasis (60.5%), peritoneal carcinomatosis (12.1%), and lymph node metastasis (68.0%). Self-expandable metallic and plastic stents were used in 64.6% and 35.4% of patients, respectively (**Table 1**).

**Table 1:** Patient characteristics and endoscopic interventions in the derivation and validation cohorts.

Characteristics	Derivation cohort (N = 339)	Validation cohort (N = 112)	*P* value
Male gender, n (%)	173 (51.0%)	64 (57.1%)	0.262
Age (years)	64.2 ± 12.4	63.4 ± 12.5	0.572
Body mass index (kg/m^2^)	21.6 ± 3.8	22.5 ± 4.7	0.037
Waiting time for ERCP (days)	19.0 (9.0–31.0)	20.5 (9.5–38.0)	0.119
**Type of malignancy**			
Cholangiocarcinoma, n (%)	181 (53.4%)	61 (54.5%)	0.844
Intrahepatic cholangiocarcinoma	31 (17.1%)	12 (19.7%)	0.624
Hilar cholangiocarcinoma	107 (59.1%)	37 (60.7%)	0.645
Extrahepatic cholangiocarcinoma	43 (23.8%)	12 (19.7%)	0.714
Pancreatic cancer	120 (35.4%)	39 (34.8%)	0.912
Gallbladder cancer	34 (10.0%)	11 (9.8%)	0.949
Malignant IPMN	4 (1.2%)	1 (0.9%)	1.000
**ECOG performance-status score, n (%)**			
1	58 (17.1%)	16 (14.3%)	0.484
2	192 (56.6%)	71 (63.4%)	0.209
3	89 (26.3%)	25 (22.3%)	0.406
**Clinical presentation, n (%)**			
Abdominal pain	194 (57.2%)	62 (55.4%)	0.729
Jaundice	303 (89.4%)	96 (85.7%)	0.292
Fever	24 (7.1%)	6 (5.4%)	0.526
Weight loss	216 (63.7%)	66 (58.9%)	0.364
Ascending cholangitis	61 (18.0%)	21 (18.8%)	0.857
**Pre-endoscopic laboratory**			
Hemoglobin (g/dL)	10.6 ± 3.0	10.8 ± 1.5	0.428
Platelet (10^9^/L)	319 (265–399)	323 (248–412)	0.724
INR	1.4 ± 0.7	1.4 ± 0.6	0.938
Total bilirubin (mg/dl)	19.2 ± 9.5	19.1 ± 10.0	0.973
Albumin (g/dL)	3.2 ± 0.6	3.2 ± 0.6	0.765
Alkaline phosphatase (IU/L)	465 (287–680)	427 (288–698)	0.783
Creatinine (mg/dl)	0.8 (0.6–1.0)	0.8 (0.6–1.0)	0.546
**Cross-sectional imaging**			
Size of obstructive tumor (cm)	3.8 (2.6–5.1)	3.6 (2.6–5.5)	0.896
Hilar obstruction, n (%)	133 (39.2%)	45 (40.2%)	0.859
Non-hilar obstruction, n (%)	206 (60.8%)	67 (59.8%)	0.859
Intrahepatic obstruction	14 (4.1%)	7 (6.3%)	0.356
Extrahepatic obstruction	192 (56.6%)	60 (53.6%)	0.571
Combined obstruction, n (%)	14 (4.1%)	6 (5.4%)	0.599
Vascular involvement, n (%)	161 (47.5%)	59 (52.7%)	0.341
Portal vein invasion, n (%)	96 (28.3%)	45 (40.2%)	0.019
Duodenal invasion, n (%)	31 (9.1%)	11 (9.8%)	0.831
Liver metastasis, n (%)	116 (34.2%)	34 (30.4%)	0.452
Distant metastasis, n (%)	205 (60.5%)	67 (59.8%)	0.903
Peritoneal carcinomatosis, n (%)	41 (12.1%)	12 (10.7%)	0.694
Lymph node metastasis, n (%)	231 (68.0%)	80 (71.4%)	0.514
**Endoscopic intervention, n (%)**			
Presence of metallic/plastic stent	219 (64.6%)/120 (35.4%)	76 (67.9%)/36 (32.1%)	0.530
One stent placement	317 (93.5%)	106 (94.6%)	0.667
One plastic stent placement	112 (33.0%)	34 (30.4%)	0.599
One metallic stent placement	205 (60.5%)	72 (64.3%)	0.472
- Uncovered SEMs	199 (58.7%)	67 (59.8%)	0.835
- Fully covered SEMs	4 (1.2%)	4 (3.6%)	0.110
- Partially covered SEMs	2 (0.6%)	1 (0.9%)	0.576
Two-stent placements	22 (6.5%)	6 (5.4%)	0.667
Two metallic stents	12 (3.5%)	4 (3.6%)	1.000
Two plastic stents	7 (2.1%)	2 (1.8%)	1.000
One metallic and one plastic stent	3 (13.6%)	0	1.000
**Post-endoscopic outcomes**			
Post-ERCP complications, n (%)	34 (10.0%)	13 (11.6%)	0.636
Stent dysfunction, n (%)	92 (27.1%)	26 (23.2%)	0.413
Stent patency time (days)	63 (28.0–105.0)	81 (40.0–159.0)	1.000
Bilirubin improvement after stenting^†^, n (%)	240 (70.8%)	81 (72.3)	0.757
Chemotherapy after ERCP, n (%)	49 (14.5%)	16 (14.3%)	0.965

Data are presented as the mean ± standard deviation, median (interquartile range), or number (proportion) of patients with a condition.

IPMN, intraductal papillary mucinous neoplasm; ECOG, Eastern cooperative oncology group; ERCP, endoscopic retrograde cholangiopancreatography; INR, international normalized ratio; SEMs, self-expandable metallic stent.

^†^Defined by total bilirubin improvement of more than 50% from baseline within 2 weeks after ERCP-guided endobiliary stent placement.

In the derivation cohort, 159 patients (46.9%) died within 90 days after endobiliary stent placement. The causes of death were acute cholangitis (7.7%), advanced-stage malignancy (5.9%), and bacterial septicemia (2.7%). Weight loss was the significant presenting symptom associated with death. Regarding pre-endoscopic laboratory findings, the non-surviving group tended to have higher TB and alkaline phosphatase values but lower serum albumin. Large tumor size and the presence of liver, peritoneal, and distant metastases on imaging were significantly associated with death (**Table 2**). Stage IV cancer based on 8^th^ edition of American Joint Committee on Cancer (AJCC) clinical staging was significantly associated with death (**Supplementary Table 1 and 2**). The self-expandable metallic stent was used more frequently than plastic stents in the surviving (58.3% vs 41.7%) and non-surviving groups (71.7% vs 28.3%). Metallic stent was used predominately in patients with more advanced metastatic disease compared with plastic stent (**Supplementary Table 3**). Patients in the surviving group had significantly longer stent patency (75.5 days vs 28 days, *P*=0.003), a higher rate of TB improvement of more than 50% from baseline within 2 weeks after stenting (77.8% vs 62.9%; *P*<0.001), and were more eligible for chemotherapy (24.4% vs 3.1%; *P*<0.001) than those in the non-surviving group. The waiting times for ERCP and post-ERCP complications of the surviving and non-surviving groups were comparable (**Table 2**). Gemcitabine plus cisplatin-based chemotherapy was used more frequent than other regimens (**Supplementary Table 4**).

**Table 2:** Baseline characteristics and endoscopic intervention among the surviving and non-surviving groups in the derivation cohort.

Characteristics	Survived (N = 180)	Deceased (N = 159)	*P* value
Male gender, n (%)	87 (48.3%)	86 (54.1%)	0.290
Age (years)	64.0 ± 12.7	64.4 ± 12.2	0.794
Body mass index (kg/m^2^)	21.7 ± 3.4	21.4 ± 4.1	0.463
Waiting time for ERCP (days)	19.0 (9.0–30.0)	20.0 (9.0–32.0)	0.468
**Type of malignancy**			
Cholangiocarcinoma, n (%)	89 (49.4%)	92 (57.9%)	0.121
Intrahepatic cholangiocarcinoma	14 (15.7%)	17 (18.5%)	0.353
Hilar cholangiocarcinoma	54 (30.0%)	53 (62.9%)	0.510
Extrahepatic cholangiocarcinoma	21 (23.6%)	22 (23.9%)	0.352
Pancreatic cancer	67 (37.2%)	53 (33.3%)	0.455
Gallbladder cancer	21 (11.7%)	13 (8.2%)	0.286
Malignant IPMN	3 (1.7%)	1 (0.6%)	0.626
**ECOG performance-status score, n (%)**			
1	34 (18.9%)	24 (15.1%)	0.355
2	105 (58.3%)	87 (54.7%)	0.503
3	41 (22.8%)	48 (30.2%)	0.122
**Multiple comorbidities** ^†^	61 (33.9%)	53 (33.3%)	0.914
**Clinical presentation**			
Abdominal pain	97 (53.9%)	97 (61.0%)	0.186
Jaundice	161 (89.4%)	142 (89.3%)	0.968
Fever	16 (8.9%)	8 (5.0%)	0.167
Weight loss	102 (56.7%)	114 (71.7%)	0.004
Ascending cholangitis	31 (17.2%)	30 (18.9%)	0.694
**Pre-endoscopic laboratory**			
Hemoglobin (g/dL)	10.7 ± 1.8	10.4 ± 4.0	0.377
Platelet (10^9^/L)	312.5 (258–393)	322 (268–404)	0.430
INR	1.3 ± 0.5	1.5 ± 0.8	0.023
Total bilirubin (mg/dl)	17.5 ± 9.7	21.1 ± 9.0	0.001
Albumin (g/dL)	3.4 ± 0.6	3.1 ± 0.6	< 0.001
Alkaline phosphatase (IU/L)	427.5 (270.0–647.5)	522.0 (309.0–716.0)	0.027
Creatinine (mg/dl)	0.8 (0.7–0.9)	0.8 (0.6–1.0)	0.515
**Cross-sectional imaging**			
Size of obstructive tumor (cm)	3.9 (2.2–3.5)	4.1 (2.8–5.8)	0.004
Hilar obstruction, n (%)	73 (40.6%)	60 (37.7%)	0.596
Non-hilar obstruction, n (%)	107 (59.4%)	99 (62.3%)	0.596
Intrahepatic obstruction	3 (1.7%)	11 (6.9%)	0.015
Extrahepatic obstruction	104 (57.8%)	88 (55.3%)	0.652
Combined obstruction, n (%)	8 (4.4%)	6 (3.8%)	0.757
Vascular involvement, n (%)	80 (44.4%)	81 (50.9%)	0.232
Portal vein invasion, n (%)	45 (25.0%)	51 (32.1%)	0.149
Duodenal invasion, n (%)	16 (8.9%)	15 (9.4%)	0.862
Liver metastasis, n (%)	49 (27.2%)	67 (42.1%)	0.004
Distant metastasis, n (%)	93 (51.7%)	112 (70.4%)	< 0.001
Peritoneal carcinomatosis, n (%)	14 (7.8%)	27 (17.0%)	0.010
Lymph node metastasis, n (%)	120 (66.7%)	111 (69.8%)	0.535
**Endoscopic intervention, n (%)**			
Length of biliary stricture (mm)	20.0 (13.0–30.0)	20.0 (15.0–30.0)	0.143
Diameter of intrahepatic biliary dilatation (mm)	16.0 ± 6.5	16.3 ± 6.5	0.225
Diameter of extrahepatic biliary dilatation (mm)	17.5 ± 6.3	17.2 ± 6.9	0.765
Presence of either metallic or plastic stent	105 (58.3%)/75 (41.7%)	114 (71.7%)/45 (28.3%)	0.010
One stent placement	168 (93.3%)	149 (93.7%)	0.888
One plastic stent placement	69 (38.3%)	43 (27.0%)	0.027
One metallic stent placement	99 (55.0%)	106 (66.7%)	0.028
- Uncovered SEMs	97 (53.9%)	102 (64.2%)	0.055
- Fully covered SEMs	1 (0.6%)	3 (1.9%)	0.345
- Partially covered SEMs	1 (0.6%)	1 (0.6%)	1.000
Two-stent placements	12 (6.7%)	10 (6.3%)	0.888
Two metallic stents	5 (2.8%)	7 (4.4%)	0.419
Two plastic stents	6 (3.3%)	1 (0.6%)	0.126
One metallic and one plastic stent	1 (8.3%)	2 (20.0%)	0.571
**Post-endoscopic outcomes**			
Post-ERCP complications, n (%)	15 (8.3%)	19 (11.9%)	0.269
Post-ERCP cholangitis	6 (3.3%)	10 (6.3%)	0.200
Post-ERCP pancreatitis	8 (4.4%)	9 (5.7%)	0.609
Duodenal perforation	0	0	–
Post-sphincterotomy bleeding	0	1 (0.6%)	0.452
Stent dysfunction, n (%)	71 (39.4%)	21 (13.2%)	< 0.001
Stent patency time (days)	75.5 (35.0–118.0)	28.0 (18.0–52.0)	0.003
Bilirubin improvement after stenting^‡^, n (%)	140 (77.8%)	100 (62.9%)	0.003
Chemotherapy after ERCP, n (%)	44 (24.4%)	5 (3.1%)	< 0.001

Data are presented as the mean ± standard deviation, median (interquartile range), or number (proportion) of patients with a condition.

IPMN, intraductal papillary mucinous neoplasm; ECOG, Eastern cooperative oncology group; ERCP, endoscopic retrograde cholangiopancreatography; INR, international normalized ratio; SEMs, self-expandable metallic stent.

^†^Defined by more than two illnesses or diseases occurring in the same person at the same time.

^‡^Defined by total bilirubin improvement of more than 50% from baseline within 2 weeks after ERCP-guided endobiliary stent placement.

### Predictors for 90-day mortality

In the univariate analysis (**Table 3**), the following were identified as significant variables associated with 90-day mortality after stent placement: intrahepatic biliary obstruction on cross-sectional imaging (*P*=0.025), peritoneal carcinomatosis (*P*=0.011), liver metastasis (*P*=0.004), distant metastasis (*P*<0.001), pre-endoscopic TB level (*P*=0.001), pre-endoscopic international normalized ratio level (*P*=0.032), size of the obstructive tumor (*P*=0.007), metallic stent (*P*=0.011), and stage of cancer (*P*=0.012). However, pre-endoscopic serum albumin (*P*<0.001), TB improvement of more than 50% from baseline within 2 weeks after stenting (*P*=0.003), and chemotherapy after ERCP (*P*<0.001) were inversely associated with 90-day mortality. In the multivariate analysis, the significant predictors of death within 90 days were the following: intrahepatic biliary obstruction (OR 5.69; 95% CI, 1.28–25.4; *P*=0.023), stage 4 cancer (OR 3.01; 95% CI, 1.81–5.02; *P*<0.001), pre-endoscopic serum albumin (OR 0.48; 95% CI, 0.32–0.74; *P*=0.001), TB improvement of more than 50% from baseline within 2 weeks after stenting (OR 0.57; 95% CI, 0.33–0.97; *P*=0.036), and chemotherapy after ERCP (OR 0.11; 95% CI, 0.04–0.31; *P*<0.001). These are shown in Model 1. These variables remained significant after an adjustment was made for the calendar year of endobiliary intervention to recognize the long recruitment period. The variables are shown in Model 2.

**Table 3:** Univariate and multivariate analyses of baseline variables for predicting 90-day mortality following endobiliary stent placement in the derivation cohort.

Univariate analysis				
Characteristics	Regression coefficient	Standard error	Unadjusted OR (95% CI)	*P* value
Intrahepatic biliary obstruction	1.478	0.661	4.39 (1.20–16.0)	0.025
Peritoneal carcinomatosis	0.886	0.349	2.43 (1.22–4.81)	0.011
Liver metastasis	0.666	0.232	1.95 (1.24–3.07)	0.004
Distant metastasis	0.802	0.229	2.23 (1.42–3.49)	< 0.001
Pre-endoscopic serum albumin level	-0.964	0.198	0.38 (0.26–0.57)	< 0.001
Pre-endoscopic total bilirubin level	0.041	0.012	1.04 (1.02–1.07)	0.001
Pre-endoscopic INR level	0.441	0.206	1.55 (1.04–2.33)	0.032
Pre-endoscopic ALP (times above ULN)	0.046	0.035	1.05 (0.98–1.12)	0.183
Endobiliary drainage with metallic stent	0.593	0.232	1.81 (1.15–2.85)	0.011
Size of obstructive tumor	0.127	0.047	1.14 (1.04–1.24)	0.007
Stage of pancreatobiliary cancer	1.102	0.440	3.01 (1.27-7.13)	0.012
Bilirubin improvement after stenting^*^	-0.725	0.243	0.48 (0.30-0.78)	0.003
Received chemotherapy after ERCP	-2.299	0.486	0.10 (0.04-0.26)	<0.001
**Multivariate analysis**				
**Characteristics**	**Regression coefficient**	**Standard error**	**Adjusted OR (95% CI)**	***P* value**
**Model 1^†^**				
Intrahepatic biliary obstruction	1.739	0.762	5.69 (1.28–25.4)	0.023
Pre-endoscopic serum albumin level	-0.729	0.217	0.48 (0.32–0.74)	0.001
Stage IV pancreatobiliary cancer	1.103	0.261	3.01 (1.81–5.02)	<0.001
Bilirubin improvement after stenting^*^	-0.569	0.272	0.57 (0.33–0.97)	0.036
Received chemotherapy after ERCP	-2.183	0.517	0.11 (0.04-0.31)	<0.001
**Model 2** ^‡^				
Intrahepatic biliary obstruction	1.567	0.771	4.79 (1.06–21.74)	0.042
Pre-endoscopic serum albumin level	-0.702	0.225	0.50 (0.32–0.77)	0.002
Stage IV pancreatobiliary cancer	1.004	0.265	2.73 (1.62–4.59)	<0.001
Bilirubin improvement after stenting^*^	-0.580	0.274	0.56 (0.34-0.96)	0.034
Received chemotherapy after ERCP	-2.218	0.529	0.11 (0.04–0.31)	<0.001

95% CI, 95% confidence interval; INR, international normalized ratio; ALP, alkaline phosphatase; OR, odds ratio; ERCP, endoscopic retrograde cholangiopancreatography.

**^†^** Model 1 included baseline factors that were significant in the univariate analysis; ^‡^Model 2 included the factors from Model 1 plus the calendar year of the endobiliary intervention.

^*^Defined by total bilirubin improvement of more than 50% from baseline within 2 weeks after ERCP-guided endobiliary stent placement.

### Development and validation of the 90-day mortality prediction model

The prediction model was developed using the significant factors associated with 90-day mortality after endobiliary stent placement. Intrahepatic biliary obstruction, stage IV cancer, pre-endoscopic serum albumin, TB improvement of more than 50% from baseline within 2 weeks after stenting, and chemotherapy after ERCP were associated with 90-day mortality. Their coefficients were 1.739, 1.103, -0.729, -0.569, and -2.183, respectively. The equation for 90-day mortality prediction was as follows:

2.091 + [1.739 x intrahepatic biliary obstruction on cross-sectional imaging (yes = 1, no = 0)] + [1.103 x stage IV cancer (yes = 1, no = 0)] + (-0.729 x pre-endoscopic serum albumin level) + [-0.569 x bilirubin improvement of more than 50% within 2 weeks after stenting (yes = 1, no = 0)] + [-2.183 x chemotherapy after ERCP (yes = 1, no = 0)], with “1” and “0” used for the presence and absence of each factor. An online score calculator is available at https://personalweb.mahidol.ac.th/Nonthalee-pau/mortality-prediction-score.

**Figure 1:**
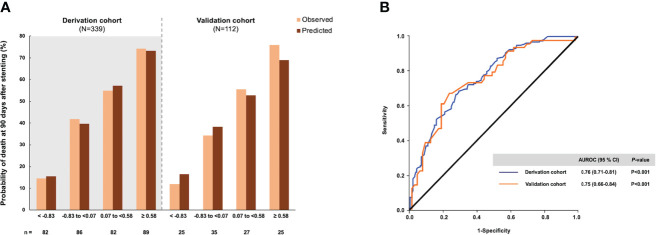
The probability of death within 90 days after endobiliary stent placement and diagnostic accuracy of the risk score in the derivation and validation cohorts. **(A)** The observed and predicted mortality rates according to the approximate quartiles of the risk score. **(B)** Area under the receiver operating characteristic curve of the risk score.

The calibration of the probability score was performed by comparing the observed and predicted 90-day probability of death according to the quartiles (**Figure 1A**). The predicted and observed probabilities of death were similar across the quartiles of the probability score in the derivation cohort (Hosmer–Lemeshow χ^2 ^= 8.00, *P*=0.434) and the validation cohort (Hosmer–Lemeshow χ^2 ^= 5.12, *P*=0.647). These results signified good performance of the probability score throughout the whole score-value range. The scores in the derivation cohort ranged from -4.94 to 4.40. The AUROC was 0.76 (95% CI, 0.71–0.81).

The validation analysis drew upon data for 112 patients with unresectable MBO. The baseline clinical characteristics, laboratory findings, and imaging findings of the validation cohort were comparable to those of the derivation cohort. The AUROC of the prediction model for 90-day mortality in the validation cohort was 0.75 (95% CI, 0.66–0.84), demonstrating the reliability of the prediction model (**Figure 1B**). The optimal cutoff of 0.03 had a sensitivity of 70.4%, specificity of 65.6%, LR+ of 2.05-2.43, and LR- of 0.43–0.45 for predicting 90-day mortality following endobiliary stent placement (**Table 4**). The high and low cutoffs were identified to categorize patients with the lowest and highest risks of death. Patients with a risk score over 1.40 were identified as a high-risk group for 90-day mortality. Of these patients, 84.2% were correctly predicted (PPVs of 84%–85%). Patients with a risk score under -1.50 were identified as a low-risk group for 90-day mortality. Eighty-nine percent of these patients were correctly predicted (NPVs of 89%–93%).

Table 4The diagnostic performance of the 90-day mortality prediction model following endobiliary stent placement in the derivation and validation cohorts.

**Table:** 

The derivation cohort						
Prediction score	Sensitivity (%) (95% CI)	Specificity (%)(95% CI)	PPV (%)(95% CI)	NPV (%)(95% CI)	LR+(95% CI)	LR-(95% CI)
Low scoreof -1.50	96.9 (92.8–99.0)	23.3 (17.4–30.2)	52.7 (50.6–54.9)	89.4 (77.3–95.4)	1.26(1.16–1.38)	0.13 (0.05–0.33)
Optimal scoreof 0.03	70.4 (62.7–77.4)	65.6 (58.1–72.5)	64.4 (59.1–69.4)	71.5 (65.9–76.6)	2.05(1.63–2.56)	0.45 (0.35–0.59)
High score of 1.40	10.1 (5.9–15.8)	96.3 (95.2–99.7)	84.2 (61.3–94.7)	55.3 (53.9–56.7)	6.04(1.79–20.34)	0.91 (0.87–0.97)

**Table:** 

The validation cohort						
Prediction score	Sensitivity (%) (95% CI)	Specificity (%)(95% CI)	PPV (%)(95% CI)	NPV (%)(95% CI)	LR+(95% CI)	LR-(95% CI)
Low scoreof -1.50	98.0 (89.2–99.9)	22.2 (12.7–34.5)	49.5 (46.0–52.9)	93.3 (65.6–99.0)	1.26(1.10–1.45)	0.09 (0.01–0.67)
Optimal scoreof 0.03	69.4 (54.6–81.8)	71.4 (58.7–82.1)	65.4 (55.1–74.4)	75.0 (65.7–82.5)	2.43(1.58–3.74)	0.43 (0.27–0.67)
High score of 1.40	12.2 (4.63–24.08)	98.4 (91.5–99.9)	85.7 (42.6–98.0)	59.1 (56.4–61.7)	7.71(0.96–61.99)	0.89 (0.80–0.99)

95% CI, 95% confidence interval; LR-, negative likelihood ratio; LR+, positive likelihood ratio; NPV, negative predictive value; PPV, positive predictive value.

The cutoffs were further explored by stratifying the prediction score into 4 intervals according to the quartiles for predicting the 90-day probability of death. In the derivation cohort, the cutoffs were as follows: scores < -0.83, scores of -0.83 to <0.07 (probability of death, 41.9%), scores of 0.07 to <0.58 (probability of death, 54.9%), and scores of ≥0.58 (probability of death, 75.2%). The prediction model was then applied to the validation cohort. The cutoffs were as follows: scores < -0.83, scores of -0.83 to <0.07 (probability of death, 34.3%), scores of 0.07 to <0.58 (probability of death, 55.6%), and scores of ≥0.58 (probability of death, 76.0%; **Table 5**).

**Table 5:** The probability of death within 90 days after endobiliary stent placement in the derivation and validation cohorts.

	Derivation cohort			Validation cohort		
Score interval	HR (95% CI)	*P* value	Probability of death	HR (95% CI)	*P* value	Probability of death
< -0.83		<0.001^†^	14.6%		<0.001^†^	12.0%
-0.83 to <0.07	3.49 (1.81–6.70)	<0.001^‡^	41.9%	3.25 (0.92–11.52)	0.068^‡^	34.3%
0.07 to <0.58	5.09 (2.69-9.63)	<0.001^‡^	54.9%	6.12 (1.77-21.19)	0.004^‡^	55.6%
≥ 0.58	8.26 (4.46–15.31)	<0.001^‡^	75.2%	12.10(3.57–41.08)	<0.001^‡^	76.0%

95% CI, 95% confidence interval; HR, hazard ratio.

^†^P value of total score (reference); ^‡^P value of each interval score compared with the reference.

### Survival analysis at 90 days

**Figure 2:**
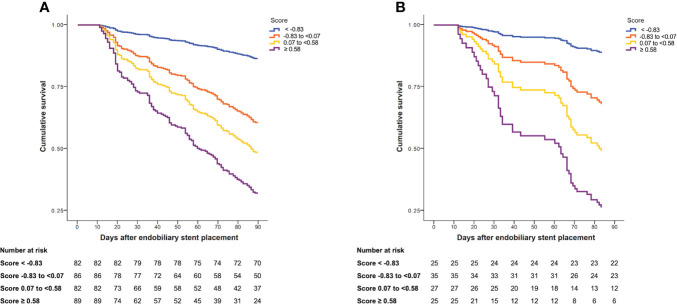
Cumulative survival at 90 days after endobiliary stent placement stratified by quartiles of risk score. **(A)** Cumulative survival in the derivation cohort. **(B)** Cumulative survival in the validation cohort.

During follow-up, 159 patients in the derivation cohort died. The median follow-up was 99 days (IQR, 47–239). The cumulative survival at 90 days after endobiliary stent placement was 52.8%. In the validation cohort, 49 patients died. The median follow-up was 114 days (IQR, 61–265). At 90 days after endobiliary stent placement, the cumulative survival was 56.3%. The cumulative survival was stratified according to the quartiles of the prediction score in the derivation and validation cohorts (**Figure 2**).

## Discussion

Biliary obstruction caused by pancreaticobiliary tumors may lead to poor outcomes, including cholangitis, delay in treatment including chemotherapy, a decreased quality of life, and higher mortality. Understanding the factors associated with mortality due to this condition will inform how to effectively allocate resources. Our study showed that pre-endoscopic serum albumin level, intrahepatic biliary obstruction, stage IV cancer, improvement of hyperbilirubinemia within 2 weeks after stenting, and receiving chemotherapy were independent risk factors for 90-day mortality in patients with pancreaticobiliary cancers.

Lower pre-endoscopic serum albumin levels were significantly associated with 90-day mortality. Albumin is a liver-synthesized plasmaprotein essential in immunomodulation, endothelium stability, and antioxidant activity (27). Hypoalbuminemia in unresectable MBO patients indicates a deterioration in liver function from long-standing biliary obstruction and has been associated with overall survival in previous studies (24, 27, 28). Hypoalbuminemia also reflects poor nutritional status due to systemic inflammation, precluding most advanced-stage MBO patients from receiving chemotherapy and contributing to a higher fatality rate. In patients with advanced liver disease, albumin levels are often low. Cirrhotic individuals were thus omitted from this investigation to minimize the potential confounding effect of pre-endoscopic albumin levels.

Intrahepatic biliary obstruction was significantly associated with 90-day mortality. Endoscopic-guided biliary decompression in obstruction at intrahepatic biliary tract portion can be challenging, especially for peripheral duct obstruction. Furthermore, patients with intrahepatic biliary obstruction frequently have a concurrent hepatic vascular invasion. This condition results in liver decompensation, liver atrophy, and small liver remnants, with endobiliary stenting failing to provide adequate biliary drainage of more than 50% of the liver volume (29). Hence, our patients with intrahepatic biliary obstruction had a greater overall mortality rate, even after performing endobiliary stenting, than those with hilar or extrahepatic biliary obstruction.

The majority of our patients with pancreaticobiliary cancers were at clinical AJCC stage IV. Using the multivariate regression analysis of clinical factors for 90-day mortality in patients with pancreaticobiliary cancers, stage IV cancer was determined as an independent risk factor for 90-day mortality after stenting. This finding is compatible with those of previous studies, which showed that liver metastases and peritoneal carcinomatosis were independent risk factors for short-term mortality in patients with pancreaticobiliary cancers (21–23, 30).

Hernandez et al. (25) found that pre-stenting TB levels of more than 14 mg/dL were substantially associated with 30-day mortality. A high pre-endoscopic TB level indicated severe, long-standing biliary obstruction and cirrhosis, contributing to poor bilirubin normalization following stenting. As a result, these individuals were ineligible for chemotherapy and had higher mortality than those with lower pre-endoscopic TB levels (31). Our study showed that a 50% improvement of hyperbilirubinemia within 2 weeks after stenting was inversely associated with 90-day mortality.

In both the surviving and non-surviving groups, metallic biliary stents were used more frequently than plastic stents. Despite the fact that metallic stent was effective for endobiliary decompression (32–36), 45.2% of our patients died within 90 days of the procedure. Notably, in our practice, metallic stents were preferred in patients with advanced disease and were anticipated to require less stent revision due to their shorter life expectancy. The findings might suggest that the use of a metallic stent in our study was indeed a mediated factor of advanced malignancy rather than a risk factor for 90-day mortality after stenting. The selection of metallic stents might be biased by endoscopists’ decisions based on their preferences, patients’ conditions, and cancer clinical staging. Therefore, the type of endobiliary stent was not included in the prediction model.

This study developed a 90-day mortality prediction model that incorporates 5 clinical predictors. The “90-day mortality prediction model” was subsequently evaluated in the validation cohort. The model displayed consistent diagnostic performance with the derivation cohort, confirming its reliability. The AUROC of the proposed 90-day mortality prediction model was 0.76, similar to the prediction model for 24-week mortality in a prior study (21). The cutoffs were determined by assessing the predictive abilities of death to provide clinical application depending on the situation. To enhance the clinical utility of the prediction model, we proposed “scores for the probability of death” that ranged from 14.6% to 75.2%, according to quartiles. Probability scores greater than 0.58 indicated a high probability (75.2%) of dying within 90 days after stenting. Combining the “90-day mortality prediction model” and the “scores for the probability of death” might guide physicians’ decisions to provide optimal palliation for patients with unresectable MBO.

For example, patients with a “90-day mortality prediction score” exceeding 1.40 are highly likely to die within 90 days following stenting. In contrast, patients with a score below -1.50 have a low chance of death. As to patients in the intermediate group (a risk score between -1.50 and 1.40), the treatment approach should be based on the patients’ performance statuses and physician discretion. Endobiliary drainage with a plastic stent is suitable for unresectable MBO patients with a high death risk (90-day mortality prediction score exceeding 1.40 and probability score over 0.58). Nonetheless, performance status, nutritional status, and comorbidities should be considered when deciding on a treatment plan. Counseling with patients and their families is needed. Prompt biliary decompression should be considered in situations of superimposed infection, such as acute cholangitis or cholangitic liver abscesses.

The strengths of this study were that it had a large cohort of unresectable MBO patients and a long data-collection duration. Furthermore, this is the first study to develop a 90-day mortality prediction model and create a validation cohort to verify the model internally. Combining the 90-day mortality prediction model and the score for probability of death enhanced the model’s performance.

Nonetheless, the current investigation has some limitations. First, it was conducted retrospectively. Consequently, some data, such as patients’ nutritional statuses, could not be evaluated, which might be associated with post-stenting mortality. Second, our analysis excluded cirrhotic patients and those with other cholestatic diseases to minimize the potential confounding effects of pre-endoscopic laboratories. Thus, this prediction model does not apply to these patients. Third, morbidity during hospitalization was not explored. This factor might affect post-stenting outcomes, such as the severity of acute cholangitis and organ failure due to sepsis or septic shock. Finally, because the unmeasured bias could occur in our study, the prediction model must be externally evaluated in larger prospective cohorts to confirm the model’s accuracy for predicting post-stenting 90 days mortality.

## Conclusions

The pre-endoscopic laboratory reflected liver performance, intrahepatic obstruction, evidence of advanced disease represented by clinical staging, improvement of hyperbilirubinemia, and eligibility for chemotherapy and were significant predictors for 90-day mortality following endobiliary stent placement. Combining a mortality prediction model and a score for probability of death can help develop an optimal drainage strategy for patients with unresectable MBO.

## Data availability statement

The original contributions presented in the study are included in the article/**Supplementary Material**. Further inquiries can be directed to the corresponding author.

## Ethics statement

The studies involving human participants were reviewed and approved by Faculty of Medicine Siriraj Hospital. Written informed consent for participation was not required for this study in accordance with the national legislation and the institutional requirements.

## Author contributions

NP contributed to the conceptualization, design, and supervision of the study. PT collected the data and organized the database. NP, PT, and PC performed the formal analyses. PT wrote the first draft of the manuscript. NP and PC reviewed and edited the manuscript. All authors contributed to the manuscript revision and read and approved the submitted version.

## Acknowledgments

The authors gratefully acknowledge Ms Khemajira Karaketklang, Department of Medicine, Faculty of Medicine Siriraj Hospital, Mahidol University for assistance with the statistical analyses. We are also indebted to Mr David Park for the English-language editing of this paper.

## Conflict of interest

The authors declare that the research was conducted in the absence of any commercial or financial relationships that could be construed as a potential conflict of interest.

## Publisher’s note

All claims expressed in this article are solely those of the authors and do not necessarily represent those of their affiliated organizations, or those of the publisher, the editors and the reviewers. Any product that may be evaluated in this article, or claim that may be made by its manufacturer, is not guaranteed or endorsed by the publisher.

## Abbreviations

AUROC, area under the receiver operating characteristic curve; ERCP, endoscopic retrograde cholangiopancreatography; INR, international normalized ratio; LR−, negative likelihood ratio; LR+, positive likelihood ratio; MBO, malignant biliary obstruction; NPV, negative predictive value; PPV, positive predictive value; SEMs, self-expandable metallic stent; TB, total bilirubin.
