# Supplementary material for: Development and validation of a 90-day mortality prediction model following endobiliary drainage in patients with unresectable malignant biliary obstruction
Source: Front Oncol. 2022 Sep 6;12:922386. doi: 10.3389/fonc.2022.922386 (PMC9487418; doi:10.3389/fonc.2022.922386)
Supplement: Supplementary file 1 [file DataSheet_1.docx]

# *Supplementary material*

# Table 1. Type of pancreatobiliary malignancy and staging among the surviving and non-surviving groups in the derivation cohort

| **Type of malignancy** | **Survived**  **(N = 180)** | **Deceased**  **(N = 159)** | ***P* value** |
| --- | --- | --- | --- |
| Cholangiocarcinoma, n (%) | 89 (49.4%) | 92 (57.9%) | 0.121 |
| Intrahepatic cholangiocarcinoma^†^ | 14 (15.7%) | 17 (18.5%) | 0.353 |
| - Stage IA-II | 3 (21.4%) | 1 (5.9%) | 0.304 |
| - Stage IIIA-IIIB | 0 | 3 (17.6%) | 0.232 |
| - Stage IV | 11 (78.6%) | 13 (76.5%) | 1.000 |
| Hilar cholangiocarcinoma^†^ | 54 (30.0%) | 53 (62.9%) | 0.510 |
| - Stage I-II | 6 (11.1%) | 3 (5.7%) | 0.489 |
| - Stage IIIA-IIIC | 21 (38.9%) | 9 (17%) | 0.012 |
| - Stage IVA-IVB | 27 (50%) | 41 (77.4%) | 0.003 |
| Extrahepatic cholangiocarcinoma^†^ | 21 (23.6%) | 22 (23.9%) | 0.352 |
| - Stage I-IIB | 4 (19%) | 2 (8.7%) | 0.403 |
| - Stage IIIA-IIIB | 6 (28.6%) | 5 (21.7%) | 0.601 |
| - Stage IV | 11 (52.4%) | 15 (65.2%) | 0.387 |
| Pancreatic cancer^†^, n (%) | 67 (37.2%) | 53 (33.3%) | 0.455 |
| Stage IA-IIB | 5 (7.5%) | 2 (3.8%) | 0.462 |
| Stage III | 32 (47.8%) | 15 (28.3%) | 0.030 |
| Stage IV | 30 (44.8%) | 36 (67.9%) | 0.011 |
| Gall bladder cancer^†^, n (%) | 21 (11.7%) | 13 (8.2%) | 0.286 |
| Stage I-IIB | 0 | 0 | - |
| Stage IIIA-IIIC | 3 (14.3%) | 1 (7.7%) | 1.000 |
| Stage IVA-IVB | 18 (85.7%) | 12 (92.3%) | 1.000 |
| Malignant IPMN^†^, n (%) | 3 (1.7%) | 1 (0.6%) | 0.626 |
| Stage IA-IIB | 2 (66.7%) | 0 | 1.000 |
| Stage III | 0 | 0 | - |
| Stage IV | 1 (33.3%) | 1 (100%) | 1.000 |

# ^†^ Pancreatobiliary clinical staging based on the 8^th^ edition of American Joint Committee on Cancer (AJCC).

# IPMN, intraductal papillary mucinous neoplasm.

# Table 2. Stage of pancreatobiliary malignancy among the surviving and non-surviving groups in the derivation cohort

| **Stage of pancreatobiliary cancer** | **Survived**  **(N = 180)** | **Deceased**  **(N = 159)** | ***P* value** |
| --- | --- | --- | --- |
| Stage I-II, n (%) | 20 (11.1%) | 8 (5.0%) | 0.042 |
| Stage III, n (%) | 62 (34.4%) | 33 (20.8%) | 0.005 |
| Stage IV, n (%) | 98 (54.4%) | 118 (74.2%) | <0.001 |

# Table 3. Baseline characteristics and endoscopic intervention among the metallic stent and plastic stent groups in the derivation cohort

| **Characteristics** | **Metallic stent**  **(N = 219)** | **Plastic stent**  **(N = 120)** | ***P* value** |
| --- | --- | --- | --- |
| Male gender, n (%) | 118 (53.9%) | 55 (45.8%) | 0.156 |
| Age (years) | 64.0 ± 12.0 | 64.7 ± 13.0 | 0.583 |
| Body mass index (kg/m^2^) | 21.5 ± 4.0 | 21.7 ± 4.0 | 0.730 |
| Waiting time for ERCP (days) | 21.0 (11.0–34.0) | 16.5 (7.0–28.0) | 0.012 |
| **Type of malignancy** |  |  |  |
| Cholangiocarcinoma, n (%) | 104 (47.5%) | 77 (64.2%) | 0.003 |
| Intrahepatic cholangiocarcinoma | 22 (10.0%) | 9 (7.5%) | 0.437 |
| Hilar cholangiocarcinoma | 63 (28.8%) | 44 (36.7%) | 0.144 |
| Extrahepatic cholangiocarcinoma | 19 (8.7%) | 25 (20.8%) | 0.001 |
| Pancreatic cancer | 91 (41.6%) | 29 (24.2%) | 0.001 |
| Gall bladder cancer | 23 (10.5%) | 11 (9.2%) | 0.695 |
| Malignant IPMN | 1 (0.5%) | 3 (2.5%) | 0.129 |
| **Stage of pancreatobiliary cancer** |  |  |  |
| Stage I-II, n (%) | 10 (4.6%) | 18 (15.0%) | 0.001 |
| Stage III, n (%) | 61 (27.9%) | 34 (28.3%) | 0.925 |
| Stage IV, n (%) | 148 (67.6%) | 68 (56.7%) | 0.046 |
| **ECOG performance-status score, n (%)** |  |  |  |
| 1 | 40 (18.3%) | 18 (15.0%) | 0.547 |
| 2 | 130 (59.4%) | 62 (51.7%) | 0.207 |
| 3 | 49 (22.4%) | 40 (33.3%) | 0.028 |
| **Multiple comorbidities**^†^ | 73 (33.3%) | 41 (34.2%) | 0.877 |
| **Clinical presentation** |  |  |  |
| Abdominal pain | 131 (59.8%) | 63 (52.5%) | 0.193 |
| Jaundice | 192 (87.8%) | 111 (92.5%) | 0.168 |
| Fever | 11 (5.0%) | 13 (10.8%) | 0.046 |
| Weight loss | 143 (65.3%) | 73 (60.8%) | 0.414 |
| Ascending cholangitis | 33 (15.1%) | 28 (23.3%) | 0.058 |
| **Pre-endoscopic laboratory** |  |  |  |
| Hemoglobin (g/dL) | 10.6 ± 4.0 | 10.4 ± 2.0 | 0.503 |
| Platelet (10^9^/L) | 322 (267–405) | 310 (254–387) | 0.275 |
| International normalized ratio | 1.4 ± 1.0 | 1.4 ± 1.0 | 0.638 |
| Total bilirubin (mg/dl) | 19.1 ± 9.0 | 19.4 ± 10.0 | 0.820 |
| Albumin (g/dL) | 3.3 ± 1.0 | 3.2 ± 1.0 | 0.285 |
| Alkaline phosphatase (IU/L) | 468.0 (283.0–698.0) | 459.0 (291.5–673.0) | 0.904 |
| Creatinine (mg/dl) | 0.8 (0.6–0.9) | 0.8 (0.6–1.0) | 0.128 |
| **Cross-sectional imaging** |  |  |  |
| Size of obstructive tumor (cm) | 4.0 (3.0–5.4) | 3.3 (2.1–4.8) | 0.003 |
| Hilar obstruction, n (%) | 81 (37.0%) | 52 (43.3%) | 0.252 |
| Non-hilar obstruction, n (%) | 138 (63.0%) | 68 (56.7%) | 0.252 |
| Intrahepatic obstruction | 11 (5.0%) | 3 (2.5%) | 0.394 |
| Extrahepatic obstruction | 127 (58.0%) | 65 (54.2%) | 0.497 |
| Combined obstruction, n (%) | 9 (4.1%) | 5 (4.2%) | 1.000 |
| Vascular involvement, n (%) | 115 (52.5%) | 46 (38.3%) | 0.012 |
| Portal vein invasion, n (%) | 69 (31.5%) | 27 (22.5%) | 0.078 |
| Duodenal invasion, n (%) | 21 (9.6%) | 10 (8.3%) | 0.701 |
| Liver metastasis, n (%) | 86 (39.3%) | 30 (25.0%) | 0.008 |
| Distant metastasis, n (%) | 146 (66.7%) | 59 (49.2%) | 0.002 |
| Peritoneal carcinomatosis, n (%) | 27 (12.3%) | 14 (11.7%) | 0.858 |
| Lymph node metastasis, n (%) | 78 (52.7%) | 33 (42.3%) | 0.137 |
| **Endoscopic intervention, n (%)** |  |  |  |
| Length of biliary stricture (mm) | 20.0 (14.0–30.0) | 20.0 (15.0–30.0) | 0.578 |
| Diameter of intrahepatic biliary dilatation (mm) | 15.5 ± 6.0 | 13.2 ± 6.0 | 0.027 |
| Diameter of extrahepatic biliary dilatation (mm) | 17.9 ± 7.0 | 16.3 ± 6.0 | 0.114 |
| **Post-endoscopic outcomes** |  |  |  |
| Post-ERCP complications, n (%) | 25 (11.4%) | 9 (7.5%) | 0.251 |
| Post-ERCP cholangitis | 11 (5.0%) | 5 (4.2%) | 0.722 |
| Post-ERCP pancreatitis | 13 (5.9%) | 4 (3.3%) | 0.294 |
| Duodenal perforation | 1 (0.5%) | 0 | 1.000 |
| Stent dysfunction, n (%) | 38 (17.4%) | 54 (45.0%) | <0.001 |
| Stent patency time (days) | 83.0 (52.0–197.0) | 43.5 (21.0–77.0) | 0.001 |
| Improvement of bilirubin after stenting^‡^, n (%) | 162 (74.0%) | 78 (65.0%) | 0.082 |
| Chemotherapy after ERCP, n (%) | 35 (16.0%) | 14 (11.7%) | 0.280 |

^†^ Defined by more than two illnesses or diseases occurring in the same person at the same time.

^‡^ Defined by at least 50% reduction of total bilirubin level within 2 weeks after ERCP-guided endobiliary stent placement.

# Table 4. Type of chemotherapy regimens among patients with pancreatobiliary malignancy in the derivation cohort

| **Chemotherapy regimens** | **Survived**  **(N = 44)** | **Deceased**  **(N = 5)** | ***P* value** |
| --- | --- | --- | --- |
| **Cholangiocarcinoma, n (%)** | 21 (47.7%) | 3 (60.0%) | 0.667 |
| Cisplatin and gemcitabine | 13 (29.5%) | 2 (40.0%) | 0.635 |
| Carboplatin and gemcitabine | 0 | 1 (20.0%) | 0.102 |
| Gemcitabine | 1 (2.3%) | 0 | 1.000 |
| Cisplatin and 5-Fluorouracil | 2 (4.5%) | 0 | 1.000 |
| Carboplatin and 5-Fluorouracil | 1 (2.3%) | 0 | 1.000 |
| 5-Fluorouracil | 3 (6.8%) | 0 | 1.000 |
| Capecitabine | 1 (2.3%) | 0 | 1.000 |
| **Pancreatic cancer, n (%)** | 20 (45.5%) | 1 (20.0%) | 0.376 |
| Cisplatin and gemcitabine | 2 (4.5%) | 0 | 1.000 |
| Gemcitabine and capecitabine | 7 (15.9%) | 0 | 1.000 |
| Gemcitabine | 6 (13.6%) | 1 (20.0%) | 0.554 |
| Gemcitabine and Erlotinib | 1 (2.3%) | 0 | 1.000 |
| FOLFIRINOX | 1 (2.3%) | 0 | 1.000 |
| Carboplatin and placitaxel | 1 (2.3%) | 0 | 1.000 |
| 5-Fluorouracil | 2 (4.5%) | 0 | 1.000 |
| **Gall bladder cancer, n (%)** | 3 (6.8%) | 1 (20.0%) | 0.359 |
| Carboplatin and gemcitabine | 2 (4.5%) | 0 | 1.000 |
| Gemcitabine and capecitabine | 1 (2.3%) | 0 | 1.000 |
| Gemcitabine | 0 | 1 (20.0%) | 0.102 |
| **Malignant IPMN, n (%)** | 0 | 0 | - |

IPMN, intraductal papillary mucinous neoplasm.
